# Relationship between perceived organizational support and professional values of nurses: mediating effect of emotional labor

**DOI:** 10.1186/s12912-022-00927-w

**Published:** 2022-06-06

**Authors:** ChaoHua Peng, Ye Chen, Tieying Zeng, Meiliyang Wu, Mengmei Yuan, Ke Zhang

**Affiliations:** 1 Department of Nursing, Tongji Hospital, Tongji Medical College, Huazhong University of Science and Technology, 1095 Jiefang Avenue, Wuhan, 430030 China; 2 School of Nursing, Tongji Medical College, Huazhong University of Science and Technology, 13 Hangkong Road, Wuhan, 430030 China

**Keywords:** Perceived organizational support, Emotional labor, Professional values, Nurse, Mediation

## Abstract

**Objectives:**

Perceived organizational support was a benefit for the work performance of nurses, which may affect emotional labor and the development of professional values. This study aimed to explore the relationship between nurses’ perceived organizational support and professional values, and investigate the mediating role of emotional labor.

**Methods:**

This was a cross-sectional study. The study was conducted in 3 tertiary hospitals in Wuhan from October 2020 to January 2021. The data were collected by a questionnaire consisting of demographic characteristics, the Emotional Labor Scale for Nurses, the nurses’ Perceived Organizational Support Scale, and the Nursing Professional Values Scale (NPVS-R). A convenience sample of 1017 nurses responded to the questionnaire survey. Pearson’s correlation analysis was used to test the relationship between variables. Predictor effects were tested using hierarchical multiple regressions. The structural equation model (SEM) was used to test the mediation effect of emotional labor on the pathway from perceived organizational support to professional values.

**Results:**

A positive moderate correlation was observed between the perceived organizational support and emotional labor (*r* = 0.524, *P* < 0.01), and a positive strong correlation was observed between perceived organizational support and professional values (*r* = 0.609, *P* < 0.01). Emotional labor and perceived organizational support were positive predictors of professional values (*B* = 0.531, 95%*CI* = 0.414 ~ 0.649; *B* = 0.808, 95%*CI* = 0.715 ~ 0.901, respectively). The association between perceived organizational support and professional values was mediated by emotional labor.

**Conclusions:**

Results showed that perceived organizational support was positively related to nurses’ emotional labor, which was in turn associated with high professional values. For nurses, improving organizational support and training nurses to engage in emotional labor through providing multiple support systems, establishing appropriate incentive mechanisms, and training nurses to regulate emotions can be effective ways to promote nurses’ professional values.

**Supplementary Information:**

The online version contains supplementary material available at 10.1186/s12912-022-00927-w.

## Background

Professional values are the framework and standard for the conduct of professionals. Any professional group has its professional values [1]. As the largest medical care group, nurses’ professional values are particularly critical, which form the basis of nursing practice and the standard for nurses to interact with patients and colleagues [2, 3]. There is a positive correlation between professional values and good professional behavior [3, 4]. The well-established and adopted professional values can help nurses improve the quality of the care, develop good relationships with patients, and promote nurses’ job satisfaction [5]. Besides, professional values affect the quality of nurses’ professional life, the degree of nurses’ burnout, and nurse retention [6]. With the increasing demand of the public for high-quality nursing services, the importance of developing nurses’ professional values has become increasingly prominent.

Relevant researches have shown that education level, job title, work stress, and organizational support could influence the nurses’ professional values [7–10]. The nurses’ professional values are negatively related to the lacking of adequate approval, insufficient perceived organizational support and adverse situation experienced in taking care of patients [11, 12]. In addition, management’s support could affect nurses’ professional commitment [13], which in turn affects values-based practice. Previous studies have also shown that perceived organizational support can help employees deal with the negative impact of inconsistent professional values [14]. Based on the previous research, we hypothesize that perceived organizational support can promote the formation of professional values.

Hypothesis 1: perceived organizational support is positively related to professional values.

Emotional labor refers to the process in which individuals express emotional behaviors in line with organizational expectations by suppressing or changing their inner feelings [15]. Nursing is highly emotional labor involved and committed work. According to the motivation theory, nurses can establish a close relationship with patients and improve patients' satisfaction through emotional labor [16, 17], making nurses feel enough approval and competence at work [18]. These positive results can promote the development of nurses’ professional values.

Hypothesis 2: emotional labor is positively related to professional values.

According to the social exchange theory and the norms of reciprocity [19], employees tend to use positive work behavior to repay the beneficial treatment from the organization. When employees receive a high level of support in customer-facing occupations, they tend to use a high level of emotional labor to reciprocate the organizational support [20]. Perceived organizational support can motivate nurses to help the organization achieve its goals. Motivated nurses tend to adjust their behaviors according to their work needs and adhere to the rules of emotional labor.

Hypothesis 3: perceived organizational support is positively related to emotional labor.

There is no standardized organizational support system in Chinese hospitals at present. But the government and hospital managers have taken some strategies that focus on providing a supportive working environment. Hospital managers make efforts to create a supportive working environment by promoting transformational leadership practices and implementing some interventions for reducing occupational stress [21, 22]. In 2008 the government published the ‘Nurse Regulations’ which emphasized ‘equal pay for equal work’, aiming to eliminate the disparities among nurses [23]. The Basic Healthcare and Health Promotion Law 2019 emphasizes that no organization or individual is allowed to endanger the personal safety of medical and health personnel [24]. These policies are all aimed at creating a better supportive working environment. For clinical nurses, perceived organizational support plays a positive role in professional values and emotional labor. In addition, through emotional labor, nurses can establish a good relationship with patients and obtain a good work experience, which positively influences nurses’ professional values. Therefore, we propose our hypothesis as follows.

Hypothesis 4: emotional labor mediates the relationship between perceived organizational support and professional values.

## Methods

### Design

The study was a cross-sectional design and adhered to the STROBE statement. A convenience sample of nurses was recruited from 3 tertiary hospitals in Wuhan, China from October 2020 to January 2021. Inclusion criteria required: (1) registered nurses, (2) willing to participate in the study. Nurses who were on vacation and came to the hospital temporarily for further study and training during data collection were excluded.

### Sample

The sample size was calculated using a single population mean formula: [*n* = Z^2^*δ^2^/ε^2^], with the assumptions of 95% confidence level (Z = 1.96), the standard deviation (δ = 11.94) [25], and maximum likely error (ε = 0.8). Considering a 5% non-response rate the final sample size becomes 899. A total of 1096 nurses submitted questionnaires. Of which, 79 questionnaires were incomplete (28 participants withdrew their consent to participate during the survey, 22 participants with missing values in demographic characteristics, 29 participants with missing values in perceived organizational support, emotional labor or professional values). Given that missing data for the key variables of perceived organizational support, emotional labor and professional values represented less than 3% of the sample, the incomplete data were treated by a complete case deletion. Therefore, 1017 nurses (92.8%) were included in the analysis.

### Procedures

The study was approved by the Clinical Trial Ethics Committee of Huazhong University of Science and Technology, Wuhan, China (NO.S161). The survey was anonymous and voluntary. Participants were informed that they were free to participate in the study or not and had the right to withdraw at any time. Only if the participants clicked through the consent form, could they gain access to the questionnaires designed on the website named Wenjuanxing, a professional online questionnaire survey, evaluation, and voting platform in China. Data collection on the Web has been used in other studies successfully [26]. This method of collecting data is efficient, reliable, and cost-effective [27].

### Instruments

#### Demographic characteristics

Demographic information was collected by a self-designed questionnaire, including gender, age, marital status, education status, professional title, and years of work.

#### The emotional labor scale for nurses

Nurses’ emotional labor was measured using the Emotional Labor Scale for Nurses, which was developed by Hong J et al. [28]. The scale has been translated and validated among Chinese nurses by Ying Y [29]. This scale includes three dimensions, namely “emotional control effort in profession” (7 items, e.g., “I try to be kind to patients genuinely from my heart”), “patient-focused emotional suppression” (5 items, e.g., “I suppress my anger when patients’ words and behaviors are unfair”), and “emotional pretense by norms” (4 items, e.g., “I exaggerate expressions of interest in patients”). Each item is rated on a five-point Likert scale ranging from 1 (not at all) to 5 (very true). Higher scores on the scale indicate higher levels of emotional labor. The scale in our study showed good internal consistency with the Cronbach’s alpha of 0.926.

#### The nurses’ perceived organizational support scale

The nurses’ Perceived Organizational Support Scale was used to measure the comprehensive perception of support from the organization [30], which had been proven to have good reliability and validity among Chinese nurses. The scale consists of 15 items and one dimension (e.g., “Organization respects my goals and values”). All items are rated on a Likert scale, from 1 (strongly disagree) to 5 (strongly agree). Higher scores on the scale indicate higher levels of perceived organizational support. The scale’s Cronbach’s alpha calculated on our data was 0.987, showing a good internal consistency.

#### The nursing professional values scale-revised

The revised form of the Nursing Professional Values Scale (NPVS-R) was devised by Weis and Schank [31]. The scale was translated into Chinese by Lin and Wang and tested for validity and reliability [32], which is the version used in the present study. The scale consists of 26 items and five dimensions: activism, social justice and human dignity, trust, professional obligations, and professionalism. All items were rated on a 5-point Likert scale from 1 (unimportant) to5 (most important). The higher the score on this scale, the higher the nursing professional values. The internal consistency reliability (Cronbach’s alpha) in Li’s study was 0.946 [33]. The scale showed good internal consistency (Cronbach’s alpha = 0.988) among nurses in our study.

### Statistical analysis

The data were analyzed by the SPSS 22.0 and Mplus 8.0. Skewness and kurtosis values for the study variables were between -2 ~ 2 indicating that the data were normally distributed [34]. Descriptive statistics were used to present participants’ demographic characteristics, such as means, standard deviations, quartile, median and proportions. The scores of perceived organizational support, emotional labor and professional values were normally distributed. Independent t-tests and ANOVA tests were used to compare the means of professional values scores across demographic characteristics. Pearson correlation analysis was used to explore the correlation among perceived organizational support, emotional labor and professional values. Hierarchical multiple linear regression analysis was adopted to examine the associated variables of the professional values. Before the regression analysis was performed, it had been confirmed not to violate the assumptions of normality, homoscedasticity and multicollinearity. The overall fit of the model was evaluated by adjusted *R*^2^ statistics, *R*^2^ change and F-test determined the significance of changes in model fit. Mediation analyses were conducted with the Mplus Version 8.0 software and analyzed the mediating effect of emotional labor between perceived organizational support and professional values. The overall fit of the model is considered well when the χ^2^ value is not significant, Comparative-of-Fit Index (CFI) and Tucker-Lewis Index (TLI) > 0.90, Root mean square error of approximation (RMSEA) and Standardized root mean squared residual (SRMR) < 0.08 [35]. A nonparametric resampling technique (bootstrapping) was used to test whether the mediating effect was significant. Bias-corrected 95% *CI* was used to assess the significance of direct and indirect effects. If the 95% *CI* did not encompass zero, the effect would be considered significant. All analyses were two-tailed.

## Results

### Participants’ demographics

The demographic characteristics of nurses are presented in Table 1. The mean age of the nurses was 31.03 years (SD = 5.65), and the median and quartile of working years were 8 (5,12). The majority of the nurses in this study were female (97.0%) and married (63.5%). 92.8% of the nurses had bachelor’s degrees or above and 55.8% were senior nurses. To explore the factors affecting professional values, a univariate analysis was performed with demographic characteristics, of which only educational status was significantly associated with professional values.

**Table 1:** Nurses’ demographic characteristics and univariate analysis related to professional values (*n* = 1017)

Variables	N(%)	professional values	
		Mean ± SD	*t/F*
Gender			-0.766
Male	31(3.0)	105.74 ± 15.605	
Female	986(97.0)	103.13 ± 18.747	
Age group (years old)			0.395
≤ 25	147(14.5)	102.40 ± 18.453	
26 ~ 30	400(39.3)	103.93 ± 17.873	
31 ~ 35	273(26.8)	102.98 ± 19.085	
> 35	197(19.4)	102.80 ± 19.790	
Marital status			0.951
Married	646(63.5)	103.41 ± 18.742	
Unmarried, widowed, divorced or separated	371(36.5)	102.23 ± 18.518	
Educational status			2.071*
College degree or under	73(7.2)	107.56 ± 18.900	
Bachelor degree or above	944(92.8)	102.88 ± 18.607	
Working years as a nurse (years)			0.352
≤ 5	341(33.5)	103.16 ± 18.035	
6 ~ 10	379(37.3)	103.85 ± 18.295	
11 ~ 15	159(15.6)	102.81 ± 19.836	
> 15	138(13.6)	102.05 ± 19.860	
Professional title			0.911
Nurse	167(16.4)	102.39 ± 18.329	
Senior nurse	567(55.8)	103.92 ± 18.378	
Supervisor nurse or above	283(27.8)	102.29 ± 19.399	

^*^P < 0.05(two-tailed)

**P < 0.01(two-tailed)

### Correlations between the study variables

Mean, standard deviations, skewness and kurtosis values for the variables and correlations between the variables are shown in Table 2. All the variables were normally distributed with skewness and kurtosis ranging from -2 to 2. The mean score of nurses’ perceived organizational support was 59.73 out of 75 points, the mean score of emotional labor was 64.28 out of 80 points, and the mean score of professional values was 103.21 out of 130 points. Perceived organizational support was positively related with emotional labor (*r* = 0.524, *P* < 0.01) and professional values (*r* = 0.609, *P* < 0.01), supporting hypothesis 1 and hypothesis 3. In addition, emotional labor was positively related to professional values (*r* = 0.502, *P* < 0.01), supporting hypothesis 2.

**Table2:** Means, standard deviations, skewness, kurtosis and correlations of variables

Variable	*Mean*	*SD*	Skewness	Kurtosis	1	2	3
1.Perceived organizational support	59.73	11.05	-0.289	-0.281	**(0.987)**		
2.Emotional Labor	64.28	8.82	-0.144	-0.504	0.524**	**(0.926)**	
3.Professional values	103.21	18.66	-0.237	-0.658	0..609**	0.502**	**(0.988)**

*N* = 1017. Cronbach’s alpha coefficients are presented in boldface on the diagonal

^*^P < 0.05(two-tailed)

^**^P < 0.01(two-tailed)

### Hierarchical multiple linear regression results

The score of professional values was used as the dependent variable for hierarchical multiple linear regression analysis. Educational status was entered in the first step, perceived organizational support was entered in the second step, emotional labor was entered in the third step, and the interaction effect of perceived organizational support and emotional labor was entered in the fourth step. The final model explained 42.1% of the variance of professional values (adjusted *R*^*2*^ = 0.421). In the first model, educational status explained 0.4% of the variance of professional values (*F* = 4.287, *P* < 0.05). The second model, in which perceived organizational support was added, explained 37.6% of the variance of professional values (*F* = 604.347, *P* < 0.001). The third model, in which emotional labor was added, explained 42.1% of the variance of professional values (*F* = 79.173, *P* < 0.001). In model 4, the moderating role of emotional labor was tested. The analysis showed that emotional labor did not moderate the relationship between perceived organizational support and professional values (*F* = 0.116, *P* > 0.05). The results showed that perceived organizational support (*β* = 0.478, *P* < 0.001) and emotional labor (*β* = 0.251, *P* < 0.001) were positive predictors of professional values. The results are presented in Table 3.

**Table3:** Hierarchical multiple linear regression analysis results of professional values

	*B*	*β*	*t*	*P*	95%*CI*(*B*)		Adjusted*R*^*2*^	*R*^*2*^ change	*F* change
					Lower	Upper			
Model 1							0.004	0.004	4.287*
Educational status^a^	-4.686	-0.065	-2.071	0.039	-9.126	-0.245			
Model 2							0.376	0.372	604.347***
Educational status^a^	-4.983	-0.069	-2.780	0.006	-8.499	-1.466			
POS	1.030	0.610	24.583	0.000	0.948	1.112			
Model 3							0.421	0.045	79.173***
Educational status^a^	-4.463	-0.062	-2.583	0.010	-7.854	-1.073			
POS	0.809	0.479	17.058	0.000	0.716	0.902			
EL	0.529	0.250	8.898	0.000	0.412	0.645			
Model 4							0.421	0.000	0.116
Educational status^a^	-4.445	-0.062	-2.570	0.010	-7.838	-1.051			
POS	0.808	0.478	17.025	0.000	0.715	0.901			
EL	0.531	0.251	8.850	0.000	0.414	0.649			
POS × EL	0.001	0.008	0.340	0.734	-0.007	0.010			

*B*: Unstandardized coefficients, *β*: standardized coefficients, *CI* confidence interval, *POS* Perceived organizational support, *EL* Emotional labor, *PV* Professional values

^a^Educational status (0 = College degree or under, 1 = Bachelor degree or above, reference group = 0)

^*^P < 0.05(two-tailed), **P < 0.01(two-tailed)

^***^P < 0.001(two-tailed)

### Analyses of the mediation effect

**Fig. 1:**
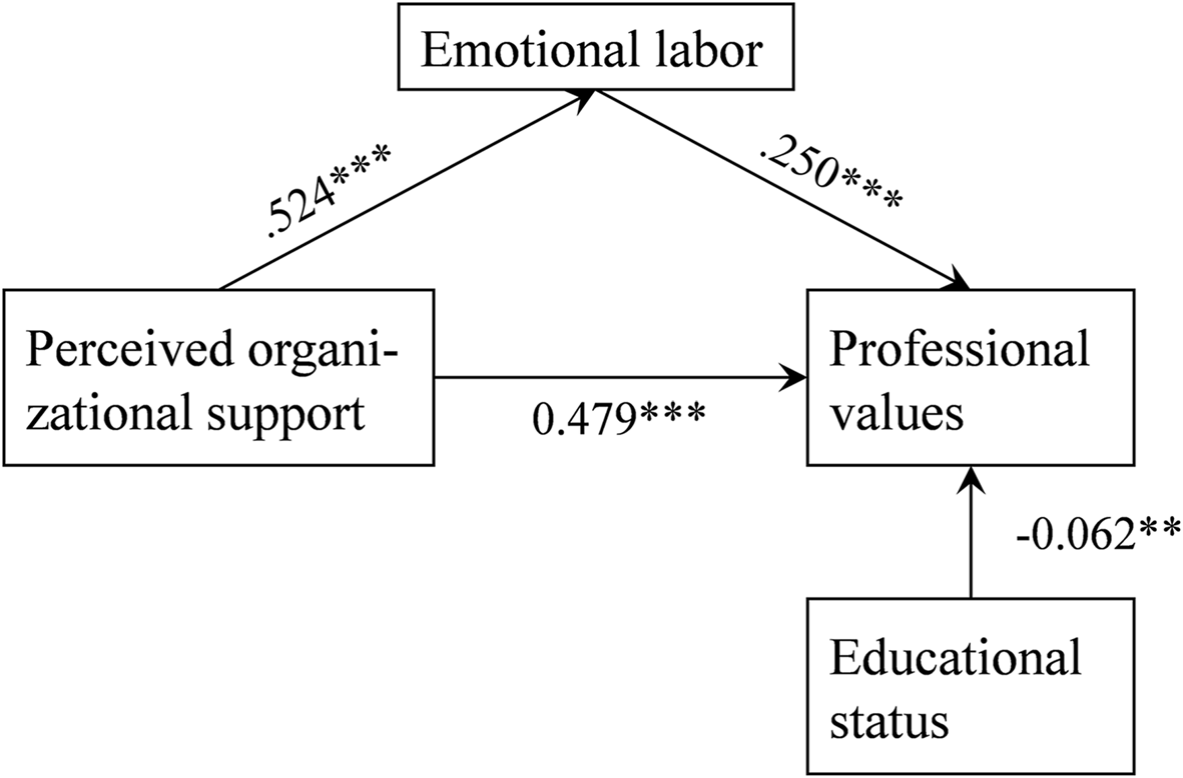
A mediation model of emotional labor in the relationship between perceived organizational support and professional values. Notes: Values are the standardized regression coefficients. * *P* < 0.05, **P < 0.01, ***P < 0.001

We developed a mediation model by taking perceived organizational support as the predictive variable, professional values as the outcome variable and emotional labor as the mediating variable. Educational status was adjusted as a covariate. Our mediation model fit the data well, with χ^2^ = 1.161 (*P* = 0.281), CFI = 1.000, TLI = 0.999, RMSEA = 0.013 (95%*CI*:0.000 ~ 0.085), SRMR = 0.010. Figure 1 shows the mediating effect of emotional labor in the association between perceived organizational support and professional values. All the paths in the model were significant (*P* < 0.05). The indirect effect of perceived organizational support on professional values through emotional labor was 0.131 (Bias-corrected 95%*CI* = 0.099 ~ 0.167), and the total effect was 0.610 (Bias-corrected 95%*CI* = 0.565 ~ 0.652). The contribution rate of the mediating effect to the total effect was 21.48%. The results indicated that emotional labor partially mediated the relationship between perceived organizational support and professional values, supporting hypothesis 4. The specific results are presented in Table 4 and Fig. 1.

**Table4:** Direct and indirect effect and 95% confidence intervals

POS → PV	Point estimate	Product of coefficients		Bootstrapping(*n* = 5000)				
				Percentile 95% *CI*		Bias-corrected 95% *CI*		*P*
		SE	Z	Lower	Upper	Lower	Upper	
Direct effect	0.479	0.030	15.751	0.694	0.921	0.417	0.535	< 0.001
Indirect effect	0.131	0.017	7.536	0.168	0.281	0.099	0.167	< 0.001
Total effect	0.610	0.023	26.880	0.931	1.126	0.565	0.652	< 0.001

Values are all standardized coefficients

*POS* Perceived organizational support, *EL* Emotional labor, *PV* Professional values, *CI* confidence interval

## Discussion

This study investigated the current status and associated factors of nurses’ professional values, as well as explored the mediating effect of emotional labor on the relationship between perceived organizational support and professional values. The results showed that perceived organizational support and emotional labor were positive predictors of professional values. In addition, emotional labor was a mediator on the pathway from perceived organizational support to professional values. These results could improve our understanding of nurses’ professional values, emotional labor and perceived organizational support, thus providing a reference to improving nurses’ professional values.

In the study, the score of NPVS-R (103.21 ± 18.66) was relatively high, which was similar to the studies in Iran and Turkey [36, 37]. The relationship between the level of education and nursing professional values was inconsistent in different studies. Some studies showed that high education levels had a positive impact on professional values [8, 38]. Some studies found no significant association between education status and professional value [39, 40]. Our study found that nurses with bachelor degrees or above obtained lower scores on professional values than nurses with college degree or under. The finding was similar to the result of a study conducted on 269 Turkish nurses [41]. Among the nurses with college degree or under, 69.7% had been working for more than 5 years and among those with bachelor degree or above, 24.7% had been working for more than 5 years. Therefore, such difference might have resulted from the nurses’ working experiences. The univariate analysis showed no significant differences between age and gender with professional values, which were consistent with the previous studies in South Korea and Taiwan [38, 42]. However, our result was different with the finding of Geckil et al. [43], who investigated 328 undergraduate nursing students and 57 clinical nurses, and found females had higher professional values. The difference in sampling structure might have contributed to the inconsistency.

Our results indicated that perceived organizational support was a positive predictor of professional values. The finding meant that better perceived organizational support contributed to a higher professional value for nurses. A good sense of perceived organizational support enables nurses to feel positive work emotions, which can promote the development of professional values [37]. In addition, emotional labor was also found to be a positive predictor of professional values. Emotional labor has both positive and negative effects on work. Previous studies on nurses’ emotional labor paid more attention to the negative impact of emotional labor, such as depression, emotional exhaustion and job burnout [44, 45] But the positive effects of emotional labor, which was beneficial to nurses’ work engagement, had also been reported [18]. Our study found that emotional labor could promote the development of nurses’ professional values. A possible explanation is that emotional labor could effectively establish a caring relationship between nurses and patients, improve the value of the nursing profession, and make nurses get enough recognition [46].

The mediation model indicated that emotional labor had a partial mediating effect on the relationship between perceived organizational support and professional values. In our study, emotional labor was positively correlated with professional values and perceived organizational support. But it is worth noting that excessive emotional labor will have negative impacts on nurses and nursing organizations if it is not recovered in time, such as threatening nurses’ physical and mental health, increasing nurses’ turnover intention and degree of burnout [47]. Based on the job demands-resources (JD-R) model [48] and the conservation of resources (COR) theory [49], the increase of work resources (such as perceived organizational support) can effectively buffer the negative impact of work demand (such as physical demands and emotional labor). As an important job resource, perceived organizational support can provide material support and spiritual support to nurses, which can promote nurses’ emotional labor, help avoid the negative effects of emotional labor, and improve nurses’ professional values. Our study proves that perceived organizational support not only has a direct association with professional values, but also has an indirect association with professional values via the mediating role of emotional labor. Therefore, we should pay more attention to promoting the development of professional values by improving perceived organizational support.

In the field of nursing, early studies paid more attention to the adverse effects of emotional labor on nurses’ physical and mental health. Our research focused on the positive effect of emotional labor on nurses’ professional value, which could enrich the theory of emotional labor in nursing. Given that emotional labor mediates the relationship between perceived organizational support and professional values, measures that contribute to regulating emotions and engaging in emotional labor may be promising for nurses to promote professional values.

The present study had several limitations. First, the data were collected in only tertiary hospitals, which might not be generalized to other contexts and limit the generalizability of results. Second, the present study was cross-sectional research that could not identify the causal relationships between variables. In subsequent research, longitudinal data should be collected to determine the causal relationships between these variables.

## Conclusion

The findings of the present study emphasized the important role of perceived organizational support and emotional labor in the development of professional values among nurses. The results suggest that nursing managers should make efforts to provide a supportive working environment for nurses to enhance the levels of perceived organizational support in order to promote their emotional labor and professional values. Meanwhile, intervention measures that motivate nurses to engage in more emotional labor, such as emotional labor skills straining and negative emotions management may be promising for promoting nurses’ professional values.

## Supplementary Information

**Additional file 1.** STROBE Statement—Checklist of items that should be included in reports of *cross-sectionalstudies.*

## Data Availability

The datasets analyzed during the current study are not publicly available due to them containing information that could compromise research participant consent but are available from the corresponding author on reasonable request.

## Abbreviations

NPVS-R: The Nursing Professional Values Scale
SEM: The Structural Equation Model
JD-R: Job Demands-Resources model
COR: The Conservation of Resources Theory
